# The Test-Retest Reliability of Heart Rate Variability and Its Association With Personality Functioning

**DOI:** 10.3389/fpsyt.2020.558145

**Published:** 2020-11-04

**Authors:** Fillip Ferreira Eikeseth, Sjur Skjørshammer Sætren, Beatrice R. Benjamin, Ingeborg Ulltveit-Moe Eikenæs, Stefan Sütterlin, Benjamin Hummelen

**Affiliations:** ^1^Section for Personality Psychiatry & Specialized Treatments, Division of Mental Health & Addiction, Oslo University Hospital, Oslo, Norway; ^2^CAMHS Sola, Division of Psychiatry, Stavanger University Hospital, Stavanger, Norway; ^3^National Advisory Unit for Personality Psychiatry, Section of Personality Psychiatry, Oslo University Hospital, Oslo, Norway; ^4^Outpatient Unit of Personality Psychiatry, Vestfold Hospital Trust, Tønsberg, Norway; ^5^Faculty of Health and Welfare Sciences, Østfold University College, Halden, Norway; ^6^Division of Clinical Neuroscience, Oslo University Hospital, Oslo, Norway; ^7^Department of Research and Innovation, Division of Mental Health & Addiction, Oslo University Hospital, Oslo, Norway

**Keywords:** heart rate variability, emotion regulation (ER), alternative model for personality disorders (AMPD), personality functioning, test-retest reliability, intraclass correlation, trait/state

## Abstract

**Background:** Heart rate variability (HRV) is a widely used non-invasive index of emotion regulation ability. The main aim of our study was to investigate the relationship between HRV and level of personality functioning in a clinical sample, most of whom had a personality disorder. Our secondary aim was to examine the test-retest reliability of HRV in our sample as there is a lack of knowledge regarding the test-retest reliability in psychiatric populations. We hypothesized that trait HRV would be negatively associated with impairments of personality functioning.

**Method:** Thirty-two adults (23 females, mean age = 27) with threshold or subthreshold personality disorders were recruited from two psychiatric outpatient clinics in Norway. Impairment of personality functioning was assessed by the first module of the Structured Clinical Interview for the DSM-5 Alternative Model for Personality Disorders (SCID-5-AMPD-I); Level of Personality Functioning Scale (LPFS). HRV was assessed during resting conditions with spontaneous breathing over three separate days. Trait HRV was calculated by averaging all three HRV assessments. The test-retest reliability of HRV was assessed using intraclass correlations.

**Results:** Contrary to expected, a positive association between trait HRV and the LPFS Self-direction domain emerged. This was driven by positive associations between the LPFS and HRV at time point 2. Overall, the test-retest reliability of HRV was comparable to previous studies on healthy subjects. However, the reliability coefficients for the first two time points were considerably lower relative to the second and third time points.

**Conclusions:** We propose that impairment of personality functioning may have increased the proportion of variance in HRV attributed to state relative to trait. This could explain the lower test-retest reliability for the first two time points. The increased test-retest reliability for the last two time points could reflect a habituation to the testing situation and hence, less pronounced influences of state in the second and third time points.

## Introduction

Heart rate variability (HRV) is a non-invasive and widely used marker of cardiac autonomic functioning. The term HRV refers to the temporal fluctuations between successive heart beats caused by respiratory cycles and primarily reflects parasympathetic (vagal) influences on cardiac activity (1). These parasympathetic influences are modulated by the central autonomic network (CAN) consisting of cortical and subcortical regions implicated in the regulation of emotional alertness, reactivity, and recovery (2, 3). Specifically, the CAN integrates a constant flow of internal and external contextual information of threat and safety and uses this to adapt the peripheral physiology (e.g., heart rate) and behavior in accordance to everchanging situational demands (1, 2). Heart rate variability may therefore be viewed as a proxy for the ability of the CAN to regulate emotional alertness, reactivity, and recovery with regards to both timing and magnitude in a given context (2). While high HRV reflects a flexible and dynamic capacity for emotional responding, low HRV on the contrary, reflects a poor integration of contextual information and a rigid adaption of behavior to situational demands.

There is robust evidence for an association between reduced HRV and psychopathology (4). In a meta-analytic comparison of both short and long term indices of resting HRV in a wider range of psychiatric disorders (i.e., mood, anxiety-related, psychotic, and substance dependence disorders) Alvares et al. (5) concluded that HRV was reduced in all patient groups compared to controls. Relatively few HRV studies exist in the field of personality disorders (PD), and most of the existing studies have concentrated on borderline PD. A meta-analytic comparison of resting HRV in individuals with borderline PD and healthy controls included five small studies whereof only two reported statistically significant differences in HRV (6). Combining these studies in the meta-analysis yielded a moderate effect size that was comparable to the findings in other psychiatric disorders (5, 6).

The traditional diagnostic systems for personality disorders have been criticized for lacking validity and reliability due to several reasons such as arbitrary diagnostic thresholds and high comorbidity rates (7, 8). A more fruitful way to move forward might be to focus on dimensions of core aspects of personality pathology, like self-regulation problems and interpersonal difficulties (9). Currently, the most well-known dimensional model is the DSM-5 Alternative Model for Personality Disorders (AMPD, (10)). In this model, dimensional aspects of personality pathology are captured by two criteria; A and B. The A criterion, or Levels Personality Functioning Scale (LPFS), aims at assessing a general severity of personality pathology conceptualized as two major components: problems in self- and interpersonal functioning. These components are specified by four areas of impairment (i.e., Identity, Self-direction, Empathy, and Intimacy), which can be assessed by the Structured Clinical Interview for the DSM-5 Alternative Model of Personality Disorders module I [SCID-5-AMPD-I; (11)]. The LPFS offers a novel approach to diagnosing personality disorders by evaluating these aspects on a severity continuum, ranging from little or no impairment of personality functioning (i.e., healthy, adaptive functioning; Level 0), to some (Level 1), moderate (Level 2), severe (Level 3), and extreme impairment of personality functioning (Level 4). Averaging these scores gives a global severity score of personality pathology, which can be used in clinical decision making or in research. The B criterion of the AMPD includes 25 pathological personality traits, organized within five higher order domains (negative affectivity, detachment, antagonism, disinhibition, and psychoticism).

The inclusion of the LPFS in the DSM-5 has given an impetus to research on the assessment of personality functioning (12), and it is to be expected that psychophysiological research will follow. According to the AMPD, emotional regulation, self-esteem regulation, and other self-regulatory capacities are closely related to interpersonal functioning, like the capacity to understand others' intentions and emotions, and the capacity to engage in close relationships. Thus, regulatory capacities are central in the conceptualization of personality functioning, which points toward HRV as an obvious candidate to study the association between personality functioning and psychophysiology (2).

To the best of our knowledge, the test-retest reliability of HRV has only been investigated in healthy subjects and non-psychiatric clinical populations such as patients with chronic heart failure (13). Consequently, there is a lack of knowledge concerning the temporal stability of HRV in psychiatric populations. Test-retest reliability refers to the temporal stability of an instrument measured over repeated occasions conducted under identical conditions (14). Intraclass correlation coefficient (ICC) is a commonly used statistic to express test-retest reliability. Coefficients above 0.5 are generally considered as moderate test-retest reliability and coefficients above 0.8 as good reliability (14). A systematic review of short-term HRV test-retest reliability studies concluded that HRV assessed during rest had moderate test-retest reliability in healthy adults (13). The highest ICCs ranged between 0.84 and 0.90 for time domain measures and between 0.67 and 0.96 for frequency domain measures (13). Later studies have reported comparable findings (15–18).

Using structural equation modeling and latent state-trait theory, Bertsch et al. (15) quantified the relative proportions of variance in HRV explained by trait and non-trait factors in repeated HRV assessments. As little as 52% of the variance in a single HRV assessment was explained by trait, but this proportion increased to 66% and 75% when aggregating two and three HRV assessments, respectively (15). Non-trait influences consist of situational factors and measurement error. Situational factors refer to variance in HRV that is not explained by stable transsituational factors (i.e., trait), thus comprising state factors. These state influences on HRV arise in part from how the individual interacts with the test situation which may be referred to as person-situation interactions. Guidelines for standardizing and conducting ECG-recordings have been recommended to reduce unsystematic measurement variance in HRV, such as allowing the participants to acclimatize to the testing environment prior to initiating the ECG recordings [e.g., (19)]. However, as individuals differ in their perception and appraisal of a given situation, there will be individual differences in the extent to which the testing situation could impact each participant. In other words, it is not possible to standardize how each individual interacts with the measurement context. Varying proportions of state variance relative to trait variance across repeated HRV assessments can potentially reduce the test-retest reliability of HRV because only variance explained by trait should be consistent across repeated measurements. As such, person-situation interactions might have implications for the test-retest reliability of HRV. Furthermore, person-situation interactions could be especially relevant in the context of personality disorders where emotional dysregulation is a central feature.

The main aim of this study was to examine the association between trait HRV and level of personality functioning as assessed by the SCID-5-AMPD-I (11) in a heterogeneous clinical sample of non-psychotic patients, most of whom had a personality disorder. We hypothesized that participants with greater impairment of personality functioning would exhibit lower trait HRV. Since knowledge about the test-retest reliability of HRV is a prerequisite for an adequate interpretation of the results, our secondary aim was to investigate the test-retest reliability of HRV in our sample.

## Methods

### Participants

Thirty-two participants (23 females) with an age range of 21–41 (mean age = 27) were recruited from psychiatric outpatient departments at Oslo University Hospital and the Hospital in Vestfold, Norway. Most participants (*n* = 25) were recruited from specialized PD treatment units at the Norwegian Network for Personality Disorders (20). Diagnostic exclusion criteria were autism spectrum disorder (ASD) and other pervasive developmental disorders, schizophrenia spectrum disorder, sequelae after brain injury, severe ongoing substance abuse, and intellectual disability. Exclusion criteria for participation in the HRV study were use of beta-blockers and extreme workout (defined as 6–7 days a week). One participant was excluded for having ASD, diagnosed after inclusion in the study, leaving 31 subjects eligible for the study.

The network units in Oslo and Tønsberg offer long-term psychodynamic therapy (2–3 years), combining group and individual therapy. All except one patient were included in the HRV project during the clinical assessment phase or during the interim period between clinical assessment and treatment. One patient was in the third year of treatment. The remaining patients (*n* = 6) were recruited from general outpatient departments and were offered treatment for depression or/and anxiety disorders.

Categorical PD diagnoses were assessed before inclusion in the study by therapists at the clinical units where the participants were recruited from, using the Structured Clinical Interview for DSM-IV Axis II PDs [SCID-II; (21)]. Diagnostic PD information of 29 participants was available. Among these, 24 participants (62%) fulfilled criteria for one or more PDs, including PD not otherwise specified. The most common PD diagnosis was borderline PD (35%), followed by avoidant PD (31%), and PD not otherwise specified (19%). Three other PD diagnoses were represented: paranoid PD (2 patients; 6.5%); dependent PD (2 patients; 6.5%), and obsessive-compulsive PD (one patient; 3.2%). Fourteen participants had one PD diagnosis and four participants had two or three PD diagnoses.

As with PD diagnoses, symptom disorders were assessed by referring clinicians, using the Mini-International Neuropsychiatric Interview (MINI) for Axis I diagnoses (22). Diagnostic information was available for 29 participants. The mean number of symptom disorders among these 29 participants was 1.8 (SD =1.6, range 0-6). Ninety-one percent had one or more symptom diagnoses, the most common being major depressive disorder (52%), followed by panic disorder (26%), and social phobia (22%).

Information about daily use of psychotropic drugs was available for 26 patients (84%). Among these, one patient used three types of psychotropics; two patients used two types; eight patients used one type, and 16 patients did not use psychotropics (62%). Antidepressants (SSRI or similar) were most commonly used (10 patients), followed by third generation antipsychotics (3 patients). One patient used a central stimulant and one patient a mood stabilizer.

### HRV Data Collection and Analysis

Electrocardiography (ECG) was recorded using portable Biopac PM150 hardware. Three active Ag/AgCl electrodes were placed on the participants' chest, using a modified Lead-II configuration: placing the negative electrode on the right clavicle, the positive on the left lowest rib, and the neutral on the right lowest rib. The hardware was connected to a portable computer containing AcqKnowledge software (Biopac-Systems, 2015) where the hardware data was graphically reproduced as a one lead ECG.

All participants were asked to refrain from nicotine and caffeine 2 h prior to the HRV assessments and received an SMS reminder the same day of the measurement. ECG was recorded in a resting state under identical conditions during a 7-min period, for which the participants were left alone in a room and placed in a comfortable chair. The participants were instructed to sit in a comfortable position, move as little as possible while breathing normally, and relax as much as possible. Inter-beat intervals (IBIs) of heart rate were retrieved via AcqKnowledge®.

Data processing and statistical analysis of HRV followed the recommendations by Malik et al. (23) and was carried out using ARTiiFACT software (24), which is based on an error detection algorithm defining individual threshold criteria for erroneously detected interbeat intervals. The data processing followed Kaufmann et al. (24) references of artifact correction by visually inspecting every signal and replacing missing or incorrect IBIs with cubic spline interpolation of neighboring intervals. Out of the 7-min ECG recordings, only the last 5-min periods were used in the data processing to exclude setting-related disturbances (experimenter leaving/entering the room). A criterion threshold was calculated for each individual recording based on the participant's distribution of IBIs to allow for further efficient identification of measurement artifacts. Erroneous beats were deleted and substituted by means of cubic spline interpolation.

Statistical time and frequency domain measures of HRV were obtained via ARTiiFACT. The root of the mean squared successive differences of R-R-intervals (RMSSD) was used as a time domain measure, and absolute high frequency (HF; 0.15–0.40 Hz) was obtained as a measure in the frequency domain. The selection of HRV indices followed recommendations by Task Force guidelines and common research practice (2, 23, 25–27).

Each participant underwent between one and three HRV assessments on separate days. Thirty-one subjects participated in the first assessment, 26 subjects participated in two out of three assessments and 18 subjects participated in all three assessments. The lower participation in the subsequent time points was due to drop-out. The measurement intervals could not be standardized due to practical reasons and were scheduled individually with each participant. The measurement intervals ranged between 1 and 113 days for T1-T2 (mean = 22), and between 1 and 75 days for T2-T3 (mean = 21).

### SCID-5-AMPD-I

The Norwegian translation of the first module of the SCID-5-AMPD-I (28) was used to assess impairment in personality functioning. The SCID-5-AMPD-I closely follows the DSM-5 AMPD, differentiating between the four elements of the LPFS, i.e., Identity and Self-direction (Self), and Empathy and Intimacy (Interpersonal). These elements are operationalized by three indicators each. In more detail, Identity contains Sense of self, Self-esteem, and Emotional dysregulation; Self-direction includes Ability to pursue meaningful goals, Constructive internal standards of behavior, and Self-reflective functioning; Empathy contains Comprehension and appreciation of others' experiences, Tolerance of differing perspectives, and Understanding of one's own behavior on others; and Intimacy comprises Depth and duration of connection with others, Desire and capacity for closeness, and Mutuality of regard reflected in interpersonal behavior. In the SCID-5-AMPD-I, each indicator is scored on a scale from 0 (no impairment) to 4 (severe impairment), and these twelve scores can be used to compute a mean score, reflecting the overall level of impairment in personality functioning, or it can be used to compute mean subscores for the four elements of the LPFS, as was done in this study. A higher score indicates more severe impairment in personality functioning.

In more detail, the SCID-5-AMPD-I starts the assessment of personality functioning by posing eight general questions to obtain a global impression of the interviewee's level of personality functioning. After these initial questions, the twelve indicators of the LPFS are assessed separately by a combination of screener questions and questions for level determination, resulting in a score varying from 0 to 4 for each indicator. Based on the interviewee's responses to these screener questions and the responses to the eight preliminary questions, the interviewer conducts a preliminary evaluation of the level at which the interviewee may be functioning, and proceeds by posing determination questions pertaining to that level. The interviewer continues to pose questions corresponding to increasing levels of impairment, until the interviewee clearly does not qualify for that level of impairment, which would imply a score just beneath that level. If none of these levels are applicable, the interviewer carries on posing questions at the level just beneath the lowest level already assessed and continues in descending order. By the end of the interview, the overall level of personality functioning is computed by dividing the total score by 12.

The SCID-5-AMPD-I was administered prior to the HRV recordings by experienced clinicians trained by Donna Bender at a two-day workshop. See Buer Christensen et al. (29) for detailed information about this training. A dual-design interrater reliability study (a video-based design and a test-retest design) conducted by the current research group found excellent intraclass correlation coefficients for both the global LPFS scores and the scores of the four elements of the LPFS (29). The participants were included in the HRV study after the administration of the SCID-5-AMPD-I. However, detailed information about the time lapse between the SCID-5-AMPD-I and the first HRV measurement is not available.

### Statistical Analysis

The statistical analyses were performed using SPSS version 25 for Windows. All variables were checked for univariate outliers and normality prior to analysis. The trait HRV variable was computed by averaging the HRV assessments of all three time points. Calculating the mean of repeated HRV assessments is an alternative way of increasing the relative proportion of trait variance without using structural equation modeling (30). For those participants who could not participate for the third HRV assessment, only the two first assessments were used. The results from the analyses are reported for both time (RMSSD) and high frequency (HF_power_) domains.

The RMSSD variables had no outlying cases and were normally distributed. The HF_power_ at T1, 2, and 3 were logarithmized (log10) to achieve normal distributions. There were no outlying scores across the LPFS domain variables.

Three intraclass correlation coefficients (ICC) based on the 3,1 formula (i.e., 2-way mixed-effects model, single measures) with absolute agreement were calculated to evaluate the test-retest reliability of the HRV assessments (31, 32). One ICC was calculated for all three HRV assessments to determine the overall test-retest reliability of the HRV assessments. Two additional ICCs were subsequently computed; one for the first and second HRV assessments and one for the second and third assessments.

## Results

The descriptive statistics are presented in Table 1. Mean LPFS was 2.0. As the threshold for a PD is at level 2 in the LPFS, this is in accordance to the observation that most participants in the sample had a PD. The results of test-retest reliability analyses are presented in Table 2. For all three measurement occasions (T1+T2+T3), the ICCs indicate good test-retest reliability, both for RMSSD and HF_power_ (0.70 and 0.67, respectively). However, the ICC estimates from T1 to T2 were considerably lower (0.50 and 0.51, respectively), representing fair agreement. It should be noted that the 95% confidence intervals were rather broad for the ICCs from T1 to T2, which can be explained by the moderate sample size in combination with large intra-individual variation from T1 to T2. Thus, there is large uncertainty in the estimation of the ICCs from T1 to T2.

**Table 1:** Descriptive statistics.

	***n***	***M (SD)***	**Range**
Age	31	27.0 (4.2)	21.0–41.0
LPFS			
Identity	31	2.4 (0.8)	0.0–3.3
Self-direction	31	2.0 (0.9)	0.0–3.3
Empathy	31	1.6 (1.0)	0.0–3.3
Intimacy	31	2.1 (1.1)	0.0–3.7
Total	31	2.0 (0.9)	0.0–3.3
RMSSD			
T1	31	47.3 (20.5)	11.8–97.3
T2	26	47.9 (22.3)	18.9–95.7
T3	18	43.8 (24.6)	8.5–88.6
Mean	26	46.3 (18.4)	13.8–81.2
HF_power_ log10			
T1	31	1,004 (731)	46–3,261
T2	26	1,283 (1,318)	102–5,813
T3	18	775 (750)	40–3,018
Mean	26	1,045 (840)	155–3,927

*M, mean; SD, standard deviation; LPFS, Level of personality functioning scale; RMSSD, root mean square of successive differences; HF, high frequency; T, time point*.

**Table 2:** Intraclass correlations for HRV.

**HRV index**	**HRV time points**	**ICC (3,1)**
RMSSD	1+2+3 (*n* = 18)	0.70 [0.47−0.86]
	1+2 (*n* = 26)	0.50 [0.15−0.74]
	2+3 (*n* = 18)	0.80 [0.54−0.92]
HF_power_ log10	1+2+3 (*n* = 18)	0.67 [0.43−0.85]
	1+2 (*n* = 26)	0.51 [0.16−0.74]
	2+3 (*n* = 18)	0.64 [0.28−0.85]

*95% Confidence intervals in brackets. HRV, heart rate variability; ICC, intraclass correlation; RMSSD, root mean square of successive differences; HF, high frequency*.

To account for the possible confounding effects of drop-out, the ICC analyses were repeated in the 18 subjects that underwent all three ECG-recordings. The estimated ICCs revealed a similar pattern of lower test-retest reliability in T1+T2 compared to T2+T3. For RMSSD, ICC for HRV T1+T2 was ICC = 0.58, 95% CIs = 0.12-0.82, *p* = 0.005, and for HRV T2+T3 ICC = 0.80, 95% CIs = 0.54-0.92, *p* < 0.001. For HF_power_, ICC for HRV T1+T2 was ICC = 0.55, 95% CIs = 0.12-0.81, *p* = 0.009, and for HRV T2+T3, ICC = 0.64, 95% CIs = 0.28-0.85, *p* =0.001. Overall ICCs (i.e., T1+T2+T3) for RMSSD was ICC = 0.70, 95% CIs = 0.47-0.86, *p* < 0.001, and for HF_power_ was ICC = 0.67, 95% CIs = 0.43-0.85, *p* < 0.001.

The results from the correlation analyses between HRV and the LPFS are presented in Table 3. At T1, the correlations between HRV and personality functioning were negative or around zero, both for RMSSD and HF_power_. At T2, these correlations were positive, and for several LPFS domains the correlations emerged as statistically significant. Specifically, for RMSSD at T2, statistically significant associations emerged for all LPFS domains except the Intimacy domain. The correlation between HRV T2 and LPFS total score and the Identity domain showed tendencies toward positive associations (*p* = 0.076 and *p* = 0.079, respectively). At T3, no statistically significant associations emerged, but there was an overall pattern of positive associations. Among these associations, RMSSD at T3 showed a tendency toward a positive association with the Self-direction domain (*p* = 0.059).

**Table 3:** Pearson correlations between HRV indices and LPFS domains.

**HRV index**	**LPFS**	**HRV T1 (*n* = 31)**	**HRV T2 (*n* = 26)**	**HRV T3 (*n* = 18)**	**HRV mean (*n* = 26)**
RMSSD	Mean LPFS	−0.121	0.448*	0.353	0.361
	Identity	−0.222	0.408*	0.257	0.270
	Self-direction	−0.051	0.503**	0.453	0.452*
	Empathy	−0.206	0.438*	0.311	0.313
	Intimacy	0.016	0.249	0.175	0.234
HF_power_ log10	Mean LPFS	−0.089	0.355	0.332	0.345
	Identity	−0.262	0.350	0.250	0.203
	Self-direction	−0.021	0.428*	0.399	0.419*
	Empathy	−0.138	0.297	0.234	0.284
	Intimacy	0.058	0.201	0.224	0.275

* sig at the .05-level. **sig at the .01-level. HRV, heart rate variability; LPFS, level of personality functioning scale; T, time point; RMSSD, root mean square of successive differences; HF, high frequency.

For mean HRV, an overall pattern of positive associations was observed with the LPFS domains, but only the association between mean HRV and Self-direction was statistically significant. In addition, mean HRV and the LPFS total score had a tendency toward a positive association in both RMSSD and HF_power_ (*p* = 0.070 and *p* = 0.084, respectively). These findings suggest that higher trait HRV was associated with higher scores on the LPFS, i.e., more impairment of personality functioning.

## Discussion

The present study investigated the associations between HRV and level of personality functioning as assessed with the SCID-5-AMPD-I. To the best of our knowledge, this is the first study to explore the relationship between HRV and personality functioning in accordance with the DSM-5 AMPD, and to assess the temporal stability of HRV in a psychiatric sample. Contrary to expected, there was a positive relationship between impairment in personality functioning and trait HRV (i.e., mean HRV over all three time points). This was driven by the positive associations between HRV at T2 and the LPFS domains. The estimated ICCs for the three HRV assessments indicated good test-retest reliability in both time and frequency domains, but the ICCs for the first two time points (T1 and T2) were considerably lower, representing fair reliability. In both the time and frequency domains, the ICCs were higher for T2+T3 compared to the ICCs for T1+T2.

As low HRV is considered as a transdiagnostic vulnerability marker for psychopathology (4), the positive associations between trait HRV and the LPFS Self-direction subscale, along with the positive associations between HRV T2 and the LPFS subscales were unexpected. A meta-analysis of resting vagal tone in individuals with borderline PD concluded that these individuals had lowered vagally-mediated HRV (6). A possible explanation for this discrepancy could be that the studies included in the meta-analysis were based on single HRV assessments. The non-significant negative correlations between HRV at T1 and the LPFS Identity and Empathy subdomains support this position. In their meta-analysis, Koenig et al. (6) point out that three out of the five included studies did not observe significant effect sizes, possibly due to lack of power. As such, although not significant, the small negative effect sizes for HRV at T1 and the LPFS Identity and Empathy subdomains may be considered in line with previous HRV research in borderline PD.

Thus, though there might be an association between low HRV and personality pathology, this association seems to be smaller in PDs than in symptom disorders. Our study even suggests that there might be no association or even a positive association between the general severity of PD and HRV. A possible explanation could be that PDs in a larger degree are influenced by psychosocial factors as compared with symptom disorders. More precisely, that PDs contain aspects that have developed under the influence of environmental factors and aspects that are based on biological factors. In fact, the inclusion of both the A (personality functioning) and B criterion (personality traits) in the DSM-5 AMPD reflects a possible differentiation between psychosocial and biological influences in the development of PDs. This assertion parallels modern conceptualizations of normal personality, which discerns between basic tendencies and characteristic adaptations (33, 34). Basic tendencies are assumed to be biologically based and are represented by the “Big Five” of normal personality, (i.e., Neuroticism, Extraversion, Openness, Agreeableness, and Conscientiousness) and has its pathological counterpart in the trait model of the AMPD. Characteristic adaptations, on the other hand, include attitudes, motives, goals, values, self-images, mental representations of significant others, and many other aspects of human individuality that are shaped by social experience. It is these aspects of personality the LPFS was designed to capture. As such, biologically based measures, including HRV, might be less sensitive to capture impairment in personality functioning according to DSM-5 since these characteristics are assumed to be more influenced by psychosocial processes than by basic biological processes. This hypothesis should be examined more extensively in future psychophysiological studies on the DSM-5 AMPD including the A criterion as well as the B criterion.

Our estimated ICCs for T2+T3 and T1+T2+T3 were comparable to previous short-term test-retest reliability studies in healthy individuals with spontaneous respiration [e.g., (13, 15, 18)]. The ICCs for T1+T2, on the contrary, were lower than in previous studies. Most studies differ in the number of days between each time point (1–210 days) but have generally reported similar test-retest reliabilities of around ICC = 0.70 (13, 15, 17, 18). As our estimated ICCs were in line with previous test-retest reliability studies in healthy individuals, it is unlikely that the variable days between each time point in our study could have confounded the test-retest reliability. This is in line with a systematic review concluding that HRV assessments recorded sequentially and 6 months apart are similarly reliable (13). In line with this, Cipryan and Litschmannova (16) did not standardize the number of days between each time point, where the two first time points occurred directly after each other while the third time point was two to 30 days after the first ECG recording. They reported ICC = 0.93 (HF_power_) for T1+T2 and ICC = 0.78 (HF_power_) for T1+T3 which is in line with previous studies using standardized intervals between time points.

Previous research has shown that up to 48% of the variance in a single HRV assessment is explained by non-trait influences such as affective states during the time of assessment (15). By aggregating repeated HRV assessments, Bertsch et al. (15) reduced non-trait influences down to 25%. Although existing guidelines for standardizing the testing environment are followed (e.g., 18), it is not possible to standardize how the individual interacts within the testing situation. This has implications for the test-retest reliability of HRV because state inherently varies across repeated measurements. As individuals with personality disorders are associated with difficulties in self and self-other representations (9) and a larger volatility of vagal activation (6) it is plausible that the state influences on HRV were exacerbated on T1 relative to T2 and T3. Our findings of relatively low ICCs for T1+T2 with an increased reliability for T2+T3 may reflect greater proportions of state relative to trait influences on the variance in T1. A major difference between T1 and the subsequent time points was the novelty of the testing situation in T1, which may have invoked greater affective responses in some participants in T1 relative to the subsequent time points. The results from the ICC analyses where the subjects that dropped out were excluded, precludes drop-out as a confounding factor in these findings. Taken together, we suggest that lower personality functioning lends the individual more vulnerable to novel situations, which in turn could exacerbate state influences on HRV, thus reducing trait variance and consequently compromising the test-retest reliability of HRV.

The findings of the present study must be viewed in light of some limitations. The moderate sample size could have contributed to type II errors. This also prevented us from considering potential confounding variables in the analyses. We therefore think that our findings should be considered as preliminary. As highlighted by Koenig et al. (6), both pharmacological and psychological treatment can have an impact on HRV. However, the effect of most types seems to be small, except for tricyclic antidepressants and clozapine (5). The majority of our sample did not use any psychotropic medication, and none used tricyclic antidepressants or clozapine. We neither deem it likely that psychological treatment had substantial effect on the results since the intervals between HRV measurements were small for most cases, and psychodynamic therapy targets long-term personality change, not immediate symptom reduction. Due to practical limitations, it was not possible to standardize the number of days between each HRV assessment in our study. However, previous test-retest reliability studies have differed in the amount of elapsed time between each assessment, and there is not convincing evidence that different time intervals affect the test-retest reliability of HRV (13). We did not assess the participants' affective states (e.g., perceived stress, anxiety, or depressive symptoms) during the HRV assessments, which we would recommend for future studies. Explicitly assessing the participants' affective states during the ECG-recordings could contribute with insight about how person-situation interactions might affect the test-retest reliability of HRV. Lastly, we did not control for habitual smoking or BMI. Despite considerable inconsistencies between HRV measures and assessment conditions, there are indications of small statistical effects of both BMI and habitual smoking on short-term HRV recordings under resting conditions [e.g., (35, 36)].

## Conclusions

Contrary to our hypothesis, impairment in personality functioning was not associated with reduced trait HRV. This discrepancy with previous studies could be explained by the fact that we measured HRV at several points, and that HRV increased substantially from the first to the second time point. Our findings show that the reliability of HRV assessed at rest with spontaneous breathing in individuals with personality disorders is comparable to previous test-retest reliability studies in healthy adults. However, the relatively low estimated ICCs for the first and second time points suggest that a higher degree of state factors compromised the reliability of the first HRV assessment. These state factors could possibly have been exacerbated by impairment in personality functioning.

## Data Availability Statement

The datasets presented in this article are not readily available because this will require a more thorough anonymization of the data and an approval by the Security officer at Oslo University Hospital. We welcome any request for the data and are open to initiate this process if necessary. Requests to access the datasets should be directed to Fillip Ferreira Eikeseth, fillip.ferreira.eikeseth@regionh.dk.

## Ethics Statement

The studies involving human participants were reviewed and approved by Regional Committee for Medical and Health Research Ethics (REK; Reference 2015/1900/REK Sørøst). The patients/participants provided their written informed consent to participate in this study.

## Author Contributions

FFE and BH: design, data collection, data analysis and manuscript preparation. SSS and BRB: design, data collection and manuscript preparation. IU-M: data collection and manuscript preparation. SSS: design, data analysis and manuscript preparation. All authors contributed to the article and approved the submitted version.

## Conflict of Interest

The authors declare that the research was conducted in the absence of any commercial or financial relationships that could be construed as a potential conflict of interest.
